# Amplicon deep sequencing of five highly polymorphic markers of *Plasmodium falciparum* reveals high parasite genetic diversity and moderate population structure in Ethiopia

**DOI:** 10.1186/s12936-023-04814-w

**Published:** 2023-12-12

**Authors:** Abeba Gebretsadik Reda, Tiffany Huwe, Cristian Koepfli, Ashenafi Assefa, Sofonias Kifle Tessema, Alebachew Messele, Lemu Golassa, Hassen Mamo

**Affiliations:** 1 Department of Microbial, Cellular and Molecular Biology, College of Natural and Computational Sciences, Addis Ababa University, Addis Ababa, Ethiopia; 2 Aklilu Lemma Institute of Pathobiology, Addis Ababa University, Addis Ababa, Ethiopia; 3 Department of Biological Sciences, Eck Institute for Global Health, University of Notre Dame, Notre Dame, USA; 4 Malaria and Neglected Tropical Diseases Research Team, Ethiopian Public Health Institute, Addis Ababa, Ethiopia; 5 Pathogen Genomics, Africa CDC, Addis Ababa, Ethiopia

**Keywords:** Genetic diversity, Population structure, *Plasmodium falciparum*, Ethiopia

## Abstract

**Background:**

*Plasmodium falciparum* genetic diversity can add information on transmission intensity and can be used to track control and elimination interventions.

**Methods:**

Dried blood spots (DBS) were collected from patients who were recruited for a *P. falciparum* malaria therapeutic efficacy trial in three malaria endemic sites in Ethiopia from October to December 2015, and November to December 2019. qPCR-confirmed infections were subject to amplicon sequencing of polymorphic markers *ama1-D3*, *csp*, *cpp*, *cpmp*, *msp7.* Genetic diversity, the proportion of multiclonal infections, multiplicity of infection, and population structure were analysed.

**Results:**

Among 198 samples selected for sequencing, data was obtained for 181 samples. Mean MOI was 1.38 (95% CI 1.24–1.53) and 17% (31/181) of infections were polyclonal. Mean H_e_ across all markers was 0.730. Population structure was moderate; populations from Metema and Metehara 2015 were very similar to each other, but distinct from Wondogent 2015 and Metehara 2019.

**Conclusion:**

The high level of parasite genetic diversity and moderate population structure in this study suggests frequent gene flow of parasites among sites. The results obtained can be used as a baseline for additional parasite genetic diversity and structure studies, aiding in the formulation of appropriate control strategies in Ethiopia.

**Supplementary Information:**

The online version contains supplementary material available at 10.1186/s12936-023-04814-w.

## Background

Malaria caused an estimated 247 million cases and 619,000 deaths worldwide in 2022 [1]. The disease is a major public health and economic problem in Ethiopia. In 2020, the Federal Ministry of Health estimated that 75% of the land mass is malarious and over 50% of the population is at risk of malaria [2]. Malaria transmission generally occurs at elevations < 2000 m and peak transmission occurs from September to December and March to May [3].

Genetic diversity of *Plasmodium falciparum* populations and multiplicity of infection (MOI) in humans might vary according to transmission intensity in different geographical regions [4–9]. The study of genetic diversity helps researchers to understand the distribution, dynamics, and genetic structure of the parasite population [10–14].

Next-generation sequencing (NGS) tools are increasingly being applied to evaluate diverse markers in the parasite genome [4, 7–10]. Compared to other targets such as antigenic markers (merozoite surface proteins and Glutamic rich proteins), single nucleotide polymorphisms (SNPs) and microsatellites, highly diverse amplicon generate more sensitive detection of minority clones in polyclonal infections [4]. This method is becoming the new gold standard in treatment efficacy studies as it has a greater ability to distinguish between new and persisting infections [11].

Amplicon sequencing studies reveal population structure and genetic relatedness among parasite populations, which can be used to understand the spread of the parasite within a region and to evaluate the effectiveness of ongoing control efforts [7, 15–22].

In this study, *P. falciparum* from northern, southern and eastern part of Ethiopia were genotyped by amplicon sequencing at 5 highly diverse markers to assess parasite diversity and malaria transmission in the country.

## Methods

### Ethics statement

The study obtained ethical approval from the Institutional Review Board (IRB) of College of Natural and Computational Sciences, Addis Ababa University, certificate reference number IRB/033/2018. Written informed consent/assent was obtained from participants or parents/guardians for minors. Malaria positive cases were treated as per the national treatment guidelines for malaria [23].

### Study area

**Fig. 1:**
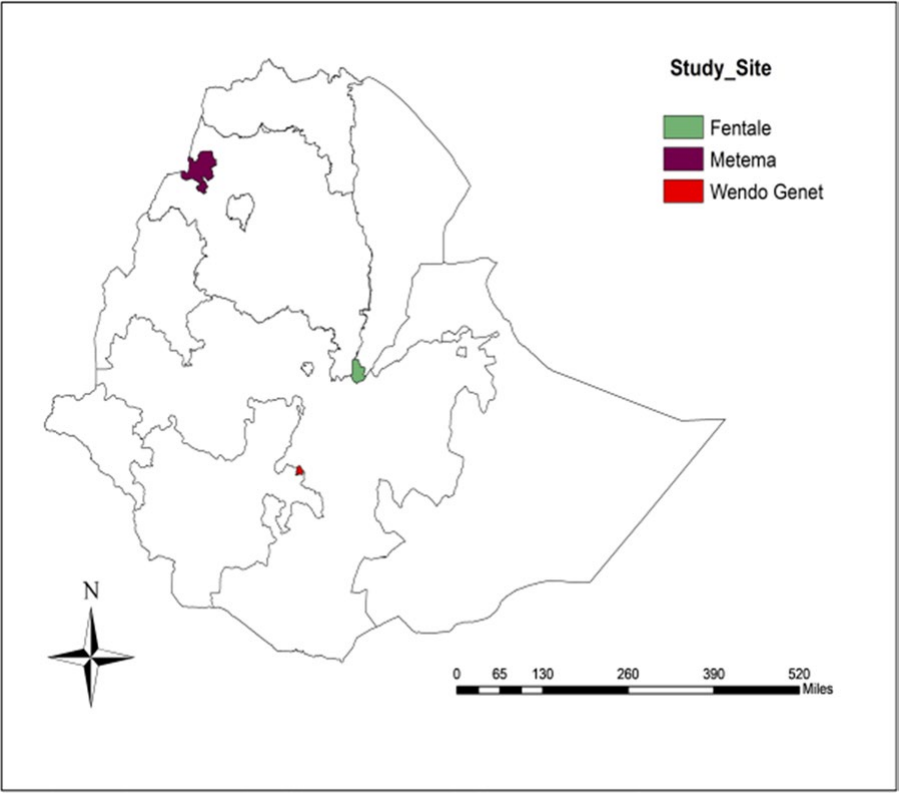
Map of the study area shows sample collection site

Samples for this study were collected in three sites: Metema, Wondogent and Metehara. Metema is in northern Ethiopia, 925 km northwest of Addis Ababa, with altitudes 1608 m above sea level River include the Genda Wuha, is a notable mosquito habitat near the study area. Wondogent in southern Ethiopia is in the Rift Valley 261 km southern of Addis Ababa at an elevation of 1,723 m. It is surrounded by primary forests and water bodies which provide suitable habitats for mosquitos. Metehara is situated in eastern-central Ethiopia in the Rift Valley area 128 km southeast of Addis Ababa at an elevation of 947 m. Nearby rivers and an irrigated sugarcane farms provide breeding sites for malaria mosquitoes (Fig. 1).

### Study population and sample collection

Study participants were febrile patients recruited from a health center in each site from October to December 2015 and again in Metehara from November to December 2019. Dried blood spots (DBS) were collected on Whatman 3 mm™ filter and stored in individual plastic bags containing desiccant.

### Laboratory methods

A piece roughly 5 mm^2^ was cut from each DBS and DNA was extracted using the NucleoMag Blood kit (Macherey–Nagel). All samples were screened by qPCR for *P. falciparum* using a *varATS* assay, targeting a multicopy gene with approximately 20 copies per genome [24]. The 3D7 *P. falciparum* culture strain was used as a positive control and to generate a standard curve for quantification. Primers and probes of qPCR are in Additional file 1: Table S1.

### Parasite genotyping

198 *Plasmodium falciparum*-positive samples with Ct values < 30 based on the *varATS* assay were selected for parasite genotyping at 5 highly diverse markers: apical membrane antigen 1 (*ama1-D3*, PF3D7_1133400), circumsporozoite surface protein (*csp*, PF3D7_0304600), a conserved *Plasmodium* protein (*cpp*, PF3D7_1475800), a conserved *Plasmodium* membrane protein (*cpmp*, PF3D7_0104100), and merozoite surface protein 7 (*msp7*, PF3D7_1335100) [12]. Amplicons were generated by PCR and samples were pooled into a sequencing library as previously described (Additional file 2: Table S2) [25]. The library was sequenced in paired-end mode in one run using the Illumina MiSeq reagent kit v3 600 cycles (2 × 300 bp) with 15% Enterobacteria phage phiX control v3 (Illumina).

### Data analysis

Haplotype calling and population analyses were conducted using R software version 4.2.2 [26] and Haplotype R package version 0.3.3 as previously described [25]. In short, low-quality reads and samples with < 10 reads were excluded. Single nucleotide polymorphisms with a mismatch rate ≥ 0.5 occurring in two or more samples were considered real. Sequences with coverage ≥ 3 and within-host frequency ≥ 1% in samples with ≥ 25 reads were considered haplotypes. A phylogenetic tree was constructed in R using *ama1-D3* major clone sequences, the K80 nucleotide substitution model, and the neighbour-joining method [27]. Principal components analysis (PCA) was conducted using the adegenet package in R. Population structure analysis was done using STRUCTURE software version 2.3.4 [28] and Bayesian clustering analysis was done with Structure harvester v0.6.94 [29].

SPSS (IBM SPSS Statistics for Windows, Version 20.0. Armonk, NY: IBM Corp.) was used to conduct additional statistical analyses. MOI was determined by the maximum number of alleles observed at an individual locus and the average MOI was calculated for each sub-population. The Student’s t-test was used to assess the relationship between MOI, parasite density, and patient age, and the spearman’s rank correlation coefficient was used to see association between age and MOIs. A chi-squared test for independence was done to compare the proportion of polyclonal infections at each site. The expected heterozygosity (H_e_) was calculated using the formula $He = \left( {\frac{n}{n - 1}} \right)\left( {1 - \sum {p^{2} } } \right),$ where ‘n’ is the number of samples analysed and ‘p’ the frequency of each allele at a locus [30].

## Results

### qPCR screening

Of a total 661 samples that were collected, 248 (38%) were collected in 2015 and 413 (62%) were collected in 2019. qPCR screening identified 280 (42%) samples positive for *P. falciparum*, 198 of which were selected based on parasite density for amplicon sequencing. Sequencing and haplotyping at two or more markers was successful for 91% (181/198) of samples (Table 1). Demographic data for 181 samples successfully sequenced is given in (Table 2).

**Table 1:** Distribution of *Plasmodium falciparum* infections among sites

Site	Samples collected	qPCR positive	Positivity (%)	High density infections (Ct < 30)	Successfully typed at 2+ markers
Metema	88	57	65	48	50
Wondogent 2015	70	57	82	48	50
Metehara 2015	90	57	63	38	33
Metehara 2019	413	109	26	61	47
Total	661	280	44	198	181

**Table 2:** Demographic, parasitological and clinical characteristics of 181 study participants with successfully sequenced *P. falciparum* infection

Characteristics	N (%)
Male/female ratio	100/81
Age (year)	Range (2–61)
Age group	
≤ 5	6 (3.3%)
6–10	8 (4.4%)
11–14	22 (12.2%)
15–19	36 (19.9%)
20–24	53 (29.3%)
≥ 25	56 (30.9%)
Temperature (°C), mean (range)	37.08 (37.01–39.07)
Geometric mean parasitemia	47.03 (95% CI 25.0–69.1)

*CI* confidence interval, *N* number of positives

### Parasite genotyping

Markers *ama1-D3*, *csp*, *cpp*, *cpmp*, and *msp7* were successfully sequenced in 177 (89.3%), 179 (90.4%), 177 (89.3%), 176 (88.9%), and 158 (79.8%) of 198 samples, respectively. All five markers were sequenced in 150/198 (75.8%) samples. Between 11 (*csp* and *msp7*) and 23 (*ama1-D3*) different alleles per marker were observed (Table 3). The median coverage for each allele in each sample (including minority clones) was 2006 reads (range 21–13,682, 2.5 and 97.5 percentiles). Among the 150 samples with sequences for all markers, 58 major clones had unique haplotypes. The most common haplotype appeared as the major clone of 13 samples, all from Metehara (3 from 2015 and 10 from 2019).

**Table 3:** Measures of genetic diversity

	Number of alleles	Polyclonal infections n/N (%)	Mean MOI	H_e_
Marker				
*AMA1-D3*	23	21/177 (11.9)	1.22	0.716
*CSP*	11	11/179 (6.1)	1.06	0.593
*CPP*	18	21/177 (11.9)	1.16	0.786
*CPMP*	20	18/176 (10.2)	1.14	0.870
*MSP7*	11	11/158 (7.0)	1.08	0.681
All markers		31/181 (17)	1.38	0.730
Site				
Metema		7/47 (14.8)	1.32	0.761
Wondogent		10/51 (19.6)	1.33	0.656
Metehara-15		8/33 (24.2)	1.67	0.775
Metehara-19		6/50 (12.0)	1.27	0.670

*He* heterozygosity index, *MOI* multiplicity of infection, *PI* polyclonal infection

MOI ranged from 1 to 7 with a mean of 1.38. The percentage of polyclonal infections was 17.5% (7/40) in Metema, 24.4% (10/41) in Wondogent, 32% (8/25) in Metehara in 2015, and 13.6% (6/44) in Metehara in 2019 and 0.716 for *ama1-D3*, 0.870 for *cpmp*, 0.786 for *cpp*, 0.593 for *csp*, and 0.681 for *msp7* (Table 3).

Mean MOI across all markers was 1.38 Detailed monoclonal and polyclonal infections by marker are given in Table 3. In 2015 18.9% (25/132) of infections were polyclonal compared to 12.2% (6/49) in 2019 (Table 3). The proportion of polyclonal infections decreased from 24 to 12% between 2015 and 2019 in Metehara; however, this trend did not reach significance (*p* = 0.48). No significant association between MOI and age or relative parasitaemia was observed (*p* > 0.05). Mean H_e_ across all sites was 0.730, indicating a moderate to high level of genetic diversity.

### Population structure

**Fig. 2:**
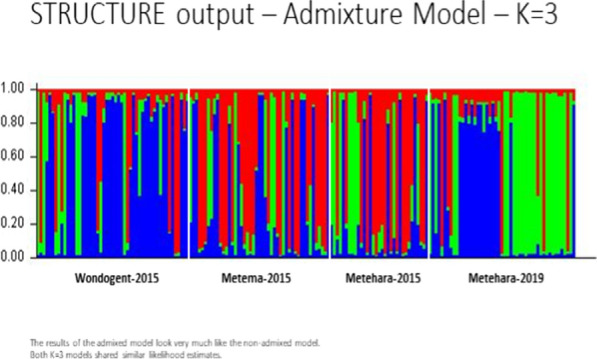
Structure plot (k = 3, admixture) showed moderate population structure. Parasites collected from Metema and Metehara-2015 shared ancestry from one group (red). Parasites from Wondogent and Metehara-2019 shared ancestry from another group (blue). Parasites with ancestry from the third group (green) were largely identified in Metehara-2019. Parasites from Metehara-2019 were dominated by two ancestral groups without much admixture (blue and green), suggesting possible clonal expansion of the two lineages

Bayesian clustering analysis of admixed indicated that the data best fit three genetic clusters. STRUCTURE analysis revealed moderate population structure among parasites. Parasites from Metema-2015 and Metehara-2015 likely share ancestry from a single genetic group. Parasites from Wondogent-2015 largely belong to a second ancestral group. Roughly 25% of parasites from Metehara-2019 share ancestry with the parasites from Wondogent, and the remaining parasites belong to a third ancestral group (Fig. 2).

**Fig. 3:**
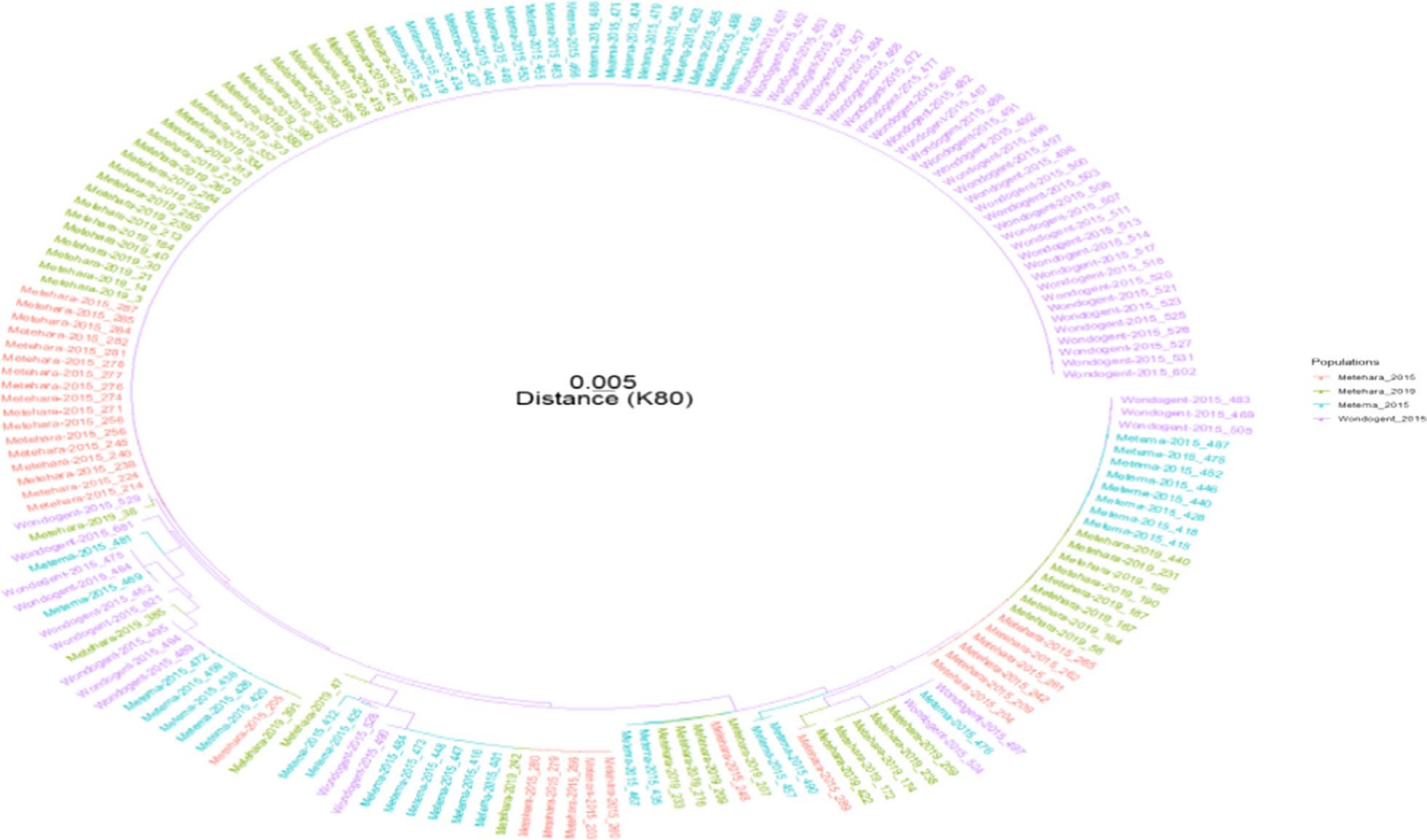
Phylogenetic tree based on *ama1-D3* alleles from major clones using neighbor-joining method. Genetic distances between parasites are small and parasites from multiple sites appear in most clades, indicating that parasites are genetically similar at this locus

**Fig. 4:**
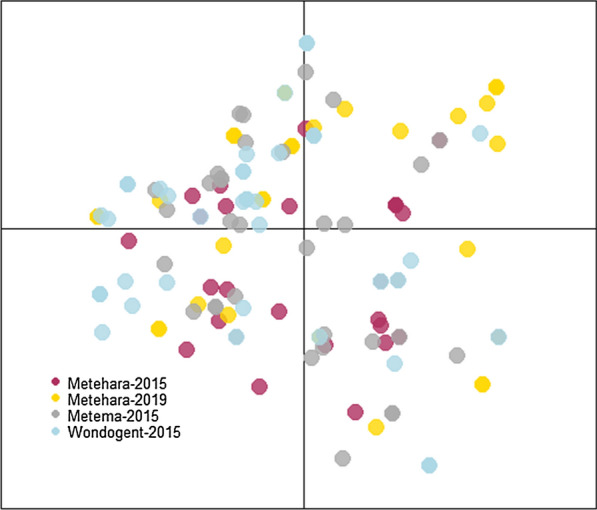
Principal components analysis considering alleles of all five markers. Parasite populations overlap, indicating gene flow and genetic similarity among groups

In a phylogenetic tree constructed based on the *ama1-D3* sequences of major clones, samples from all sites were intermixed. No clades were unique to a single study site, suggesting a high level of genetic relatedness (Fig. 3). PCA considering alleles of all five markers showed that there is more diversity within parasite populations than between them as the samples are widely scattered yet overlap almost entirely (Fig. 4). This indicates gene flow among the three sites, and that populations did not change much from 2015 to 2019 in Metehara.

## Discussion

Like many African countries, Ethiopia is working toward malaria elimination to alleviate the burden of the disease [1]. Molecular studies on *P. falciparum* genetic diversity and population structure can aid in monitoring the impact of different intervention strategies. This study employed amplicon sequencing to characterize parasite genetic diversity and structure across three malaria-endemic regions in Ethiopia. This study found moderate population structure and high genetic diversity among parasites with little distinction between parasites collected from the various study sites, likely due to gene flow among sites. High diversity of multi-locus haplotypes is typical in regions of high malaria transmission, as mosquitoes are more likely to take up multiple *P*. *falciparum* clones during a blood meal, thereby increasing the chance of recombination between unidentical parasites and generating a highly diverse parasite population [12, 14].

In this study, H_e_ among all samples was 0.73. This is similar to previous H_e_ measurements in Kenya, Nigeria, and Ghana [21, 25, 31], and lower than H_e_ values reported in Asia [25, 32]. The H_e_ reported here is substantially greater than those reported in other recent Ethiopian studies (0.17, 0.47, 0.54) [33–35]. This highlights the importance of noting methodological differences before comparing study results. Findings in this study of 17% polyclonal infections and mean MOI of 1.38 were in alignment with other recent findings in Ethiopia [33–35].

The isolates of *P. falciparum* analysed in this study were genetically diverse and generally did not cluster by collection site. This is indicative of gene flow among the geographic locations sampled, likely due to immense human traffic through the country as part of routine socio-economic activities. Additionally, this study revealed evidence of a shift in parasite populations between 2015 and 2019 in Metehara. Between the two time points MOI, heterozygosity, and the proportion of polyclonal infections decreased, although the differences were not statistically significant. Structure analysis showed a shift from a diverse parasite population arising from three ancestral groups in 2015 to a more structured population comprising just two ancestral groups in 2019. Additionally, this decrease in genetic diversity may represent a decrease in malaria transmission or a reduction in gene flow to and from Metehara by 2019. In this study, there is a reduction of MOI similar to the study done in Indonesia 1.6 [36] and a study done in Senegal 1.35 [37]. In this study, there is also a high level of heterozygosity, similar to the study done in Ghana [38].

The low to moderate level of genetic differentiation of parasites between the study sites is likely a consequence of immense human traffic as part of routine socio-economic activities. Further sampling of more populations along this North, South and East of Ethiopia including asymptomatic infections, will refine the boundaries of gene flow and inform the Ethiopia National Malaria Control Programme (NMCP) on local approaches to tackle malaria elimination.

## Conclusion

This study employed amplicon sequencing of five polymorphic markers to characterize *P. falciparum* diversity and structure across malaria-endemic sites in Ethiopia. Overall, parasites were highly diverse, with little distinction by collection site. This is indicative of gene flow among parasite populations around the country. Further sampling of more populations along this North, South and East of Ethiopia including asymptomatic infections, will refine the boundaries of gene flow and inform the Ethiopia National Malaria Control Programme (NMCP) on local approaches to tackle malaria elimination. Therefore the study will help as baseline for further study.

### Supplementary Information

**Additional file 1: Table S1.** Primers and probe used for qPCR assay.

**Additional file 2: Table S2.** Detailed procedure used for deep amplicon sequencing of five highly polymorphic markers.

## Data Availability

The dataset supporting the conclusion of this article is included within the article and the primers used to genotype the five genes are presented in attached Additional file 2: Table S2.

## Abbreviations

*ama1-D3*: Apical membrane protein1
*cpp*: Conserved *Plasmodium* protein
*csp*: Circumsporozoite surface protein
*cpmp*: Conserved *Plasmodium* membrane protein
DBS: Dry blood spot
H_e_: Expected heterozygosity
MOI: Multiplicity of infection
*msp7*: Merozoite surface protein 7
NGS: Next generation sequencing
PCA: Principal components analysis
PCR: Polymerase chain reaction
qPCR: Quantitative polymerase chain reaction
SNP: Single nucleotide polymorphism
WHO: World Health Organization
