# Supplementary material for: Amplicon deep sequencing of five highly polymorphic markers of Plasmodium falciparum reveals high parasite genetic diversity and moderate population structure in Ethiopia
Source: Malar J. 2023 Dec 12;22:376. doi: 10.1186/s12936-023-04814-w (PMC10714478; doi:10.1186/s12936-023-04814-w)
Supplement: Supplementary file 1 — Additional file 1: Table S1. Primers and probe used for qPCR assay. [file 12936_2023_4814_MOESM1_ESM.docx]

Additional file 1: Table S1. Primers and probe used for qPCR assay

| Target Gene | Oligo sequence | Fluorophores | TM °C | Number of Cycle |
| --- | --- | --- | --- | --- |
| Pspp18S F | GCTCTTTCTTGATTTCTTGGATG |  | 50° 2 min | 45 cycles |
| Pspp18S R | AGC AGG TTA AGA TCT CG TTC G |  | 95° 2 min |  |
| Pspp18S Cy5 | ATG GCC GTT TTT AGT TCG TG | Cyanine 5 | 95° 10 sec |  |
| PfvarATS F | CCCATACACAACCAAYTGGA |  | 55° 30 sec |  |
| PfvarATS R | TTCGCACATATCTCTATGTCTATCT |  | 52.76 |  |
| PfvarATS FAM | TRTTCCATAAAGGT 5ʹ-3ʹ | Fluorescein | NA |  |
