# Supplementary material for: Amplicon deep sequencing of five highly polymorphic markers of Plasmodium falciparum reveals high parasite genetic diversity and moderate population structure in Ethiopia
Source: Malar J. 2023 Dec 12;22:376. doi: 10.1186/s12936-023-04814-w (PMC10714478; doi:10.1186/s12936-023-04814-w)
Supplement: Supplementary file 2 — Additional file 2: Table S2. Detailed procedure used for deep amplicon sequencing of five highly polymorphic markers. [file 12936_2023_4814_MOESM2_ESM.docx]

Additional file 2: Table S2. Detailed procedure used for deep amplicon sequencing of five highly polymorphic markers

| 1. **DNA extraction**    1. NucleoMag Blood kit (Macherey-Nagel) following manufacturer’s instructions    2. <https://www.mn-net.com/media/pdf/32/a9/70/Instruction-NucleoMag-Blood-200.pdf> |
| --- |
| 1. **Screen for Pf**   *P. falciparum* varATS qPCR  Reference: Ultra-sensitive detection of Plasmodium falciparum by amplification of multi-copy subtelomeric targets.  Hofmann N, Mwingira F, Shekalaghe S, Robinson LJ, Mueller I, Felger I  PLoS Medicine 2015  Prepare primers and probe to **10 uM**  varATS forward+reverse primer mix 0.48 uL  varATS_probe 0.48 uL  FastAdvanced MM 2x 6 uL  H2O 1.04 uL  DNA 4 uL  Total 12 uL  varATS forward cccatacacaaccaaytgga  varATS reverse ttcgcacatatctctatgtctatct  varATS probe 6-FAM-trttccataaatggt-NFQ-MGB  50° 2 min  95° 2 min  95° 10 sec  55° 30 sec => 45 cycles |
| 1. **Select high density samples for amplicon sequencing**    1. 198 samples that were Pf positive with Ct < 30 |
| 1. **Primary PCR**    1. 2 PCRs per sample       - ama1-D3/cpmp in one reaction       - cpp/csp/msp7 in one reaction   Reference:  Amplicon deep sequencing improves Plasmodium falciparum genotyping in clinical trials of antimalarial drugs.  Gruenberg A, Lerch A, Beck HP, Felger I.  Scientific Reports 2019.  Prepare primer mixes to **10 uM**  KAPA HiFi HotStart ReadyMix 2x 7.5 uL  Primer pair 1 (fw/rv) 0.375 uL  Primer pair 2 (fw/rv) 0.375 uL  Primer pair 3 (fw/rv) or H2O 0.375  H2O 3.375 uL  DNA 3 uL  **Total 15 uL** |
| **Primers for primary PCR**  ama1-D3_prim_fw GTTTAATTAACAATTCATCATAC  ama1-D3_prim_rv GTGTTGTATGTGATGCTC  cpmp_prim_fw_new ATATGGATTGTTATAATGAAACG  cpmp_prim_rv_new CGTTACTATCAAGATCGTTAATAT  cpp_prim_fw TGTCTGAACCAAATTCAA  cpp_prim_rv GAATTTGTCACATTTGATGA  csp_prim_fw ATCAAGGTAATGGACAAG  csp_prim_rv ACTCAAACTAAGATGTGTTC  msp7_prim_fw GTATTATCAAAGGTAAAGGCA  msp7_prim_rv TTGCATAACTATAAACACCAT  95° 3 min  98° 20 sec  **x**° 15 sec **x**= 54° for cpp/csp/msp7, 52° for ama1-D3/cpmp  72° 45 sec => 20 cycles  72° 2 min |
| **5.** Nested PCR  a. 5 PCRs per sample, one reaction for each marker  Reference: Amplicon deep sequencing improves Plasmodium falciparum genotyping in clinical trials of antimalarial drugs.  Gruenberg A, Lerch A, Beck HP, Felger I.  Scientific Reports 2019.  Prepare primer mixes to 10 uM  KAPA HiFi HotStart ReadyMix 2x 7.5 uL  Primer pair (fw/rv) 1.25 uL  H2O 5.25 uL  Template (product of primary PCR) 1 uL  Total 15 uL  Primers for nested PCR  ama1-D3_fw_linker GTGACCTATGAACTCAGGAGTCTACTACTGCTTTGTCCCATC  ama1-D3_rv_linker CTGAGACTTGCACATCGCAGCTCAGGATCTAACATTTCATC  cpmp_fw_linker_new GTGACCTATGAACTCAGGAGTCATATGGATTGTTATAATGAAACG  cpmp_rv_linker_new CTGAGACTTGCACATCGCAGCCGTTACTATCAAGATCGTTAATAT  cpp_fw_linker GTGACCTATGAACTCAGGAGTCCAAGTTCACTTTTGGGAAATG  cpp_rv_linker CTGAGACTTGCACATCGCAGCATTACTACCTTTCAGCATATCCGA  Csp_fw_linker GTGACCTATGAACTCAGGAGTCAAATGACCCAAACCGAAATGT  csp_rv_linker CTGAGACTTGCACATCGCAGCGGAACAAGAAGGATAATACCA  msp7_fw_linker GTGACCTATGAACTCAGGAGTCATGAACAAGAGATATCAACACA  msp7_rv_linker CTGAGACTTGCACATCGCAGCTTAAATTGTTCATGGTATTCCTTA  95° 3 min  98° 20 sec  55° 15 sec  72° 45 sec => 10 cycles  98° 20 sec  62° 15 sec  72° 45 sec => 10 cycles  72° 90 sec |
| **6.Pool nested PCR products for each sample**   - 1. Combine equal volumes of nested PCR products from each marker into one pool for each sample      - e.g. 5uL from each marker * 5 markers = 25uL pooled product |
| 1. **Adapter/Barcode PCR**    1. One PCR per sample to attach barcode and adapter sequences to amplicon    2. Use a unique combination of forward and reverse primers for each sample       1. e.g. Fw_1 + Rv_1 for Sample1, Fw_1 + Rv_2 for Sample2   Reference: Amplicon deep sequencing improves Plasmodium falciparum genotyping in clinical trials of antimalarial drugs.  Gruenberg A, Lerch A, Beck HP, Felger I.  Scientific Reports 2019.  Prepare primer mixes to **10 uM**  KAPA HiFi HotStart ReadyMix 2x 7.5 uL  Primer fw 1.25 uL  Primer rv 1.25 uL  H2O 4 uL  Template (pooled product of nPCRs) 1 uL  **Total 15 uL**  95° 3 min  98° 20 sec  58° 30 sec  72° 45 sec => 10 cycles  72° 2 min |
| **Primers for Adapter PCR (XXXXXXXX=barcode)**  Forward AATGATACGGCGACCACCGAGATCTACACTCTTTCCCTACACGACGCTCTTCCGATCT**XXXXXXXX**GTGACCTATGAACTCAGGAGTC  Reverse CAAGCAGAAGACGGCATACGAGATCGGTCTCGGCATTCCTGCTGAACCGCTCTTCCGATCT**XXXXXXXX**CTGAGACTTGCACATCGCAGC  **Forward barcode Reverse barcode**  F1 GCAACTGT R1 TCCGATCT  F2 GAAGTACC R2 ACGAGAGA  F3 GCCTTGAT R3 ATCTGTCC  F4 CCAGGTTA R4 TGCTGCAA  F5 GCTGACAA R5 CCACTAAG  F6 TAGTGACG R6 TACCTTGC  F7 GAGTTCGA R7 CGATCACA  F8 AGAACCAC R8 ACCAAGTG  F9 CTCCTCTA R9 CGGAAGAA  F10 TTAACGCG R10 AATGGTGG  F11 GGCATTCA R11 GGTTCCTT  F12 CACTCTAG R12 GGACATTG  F13 ATACAGGC R13 TCATACGG  F14 AGTCGATC R14 CGTTATGC  F15 TCTAGGAC R15 CTGGCATT  F16 CACGAAGA R16 ACTCTGCA  F17 AAGCACAG R17 GAACGGAA  F18 AGCTTAGG R18 TTGGTCAC  F19 CATAGCCA R19 GTGAACCT  F20 TGGCAAGT R20 ATCGCGAA |
| 1. **Pool samples by concentration**    1. Run barcode PCR product on a gel.    2. Rate the intensity of the band for each sample from 0 (no band) to 3 (most intense band).    3. Create 4 pools of samples with roughly equal band intensity by mixing 4uL from each sample.       1. e.g. Mix 4uL from each sample with intensity 0 into a 0 pool.   Mix 4uL from each sample with intensity 1 into a 1 pool, etc. |
| 1. **Purify pooled PCR products**    1. Purify each pool using Ampure beads (Beckman Coulter) following manufacturer’s instructions.    2. <https://www.beckmancoulter.com/wsrportal/techdocs?docname=B37419> |
| 1. **Sequencing**    1. Pools were diluted to 0.1ug/uL and combined to form the sequencing library.    2. Library was sequenced in paired end-mode       - Illumina MiSeq reagent kit v3 600 cycle (2 × 300 bp) with 15% Enterobacteria phage phiX control v3 |
